# Comparing the Efficiency of Infrazygomatic Crest (IZC) Screws and Conventional Method for Anterior Retraction in Patients Undergoing Fixed Orthodontic Treatment for Class 2 Malocclusion: A Prospective Clinical Study

**DOI:** 10.7759/cureus.54599

**Published:** 2024-02-21

**Authors:** Sandeep Shetty, Abirami Ramesh, Salwa B Maniyankod, Katheesa Parveen, Stanly G Selvakumar, Minaz Mubeen, Vivek Amin

**Affiliations:** 1 Orthodontics and Dentofacial Orthopaedics, Yenepoya Dental College, Mangalore, IND

**Keywords:** class 2 malocclusion, anterior teeth retraction, distalization, infrazygomatic crest implants, fixed orthodontic treatment

## Abstract

Introduction

In orthodontic treatment for class 2 malocclusion, conventional approaches involve extracting the upper first premolars and using methods like en masse retraction and extra-oral or intra-oral distalization. However, these often result in unintended forces and adverse effects. Contemporary techniques, such as maxillary arch distalization with mini-implants like infrazygomatic crest (IZC) implants, offer superior outcomes. IZC implants provide a safe, flexible, and effective site for implant placement, achieving a remarkable 93.7% success rate. Power arms enable precise control, allowing orthodontists to apply controlled forces for optimal tooth movement. This study aims to compare cephalometric parameters pre and post treatment using IZC/buccal shelf (BS) screws and conventional retraction, assessing the efficiency of IZC screws in maintaining arch length during teeth retraction.

Methods

In a split-mouth study at Yenepoya Dental College, 40 orthodontic patients aged 18-35 were divided into control (premolar extraction, anterior retraction) and study (third molar removal, IZC screw distalization) groups. The control group used a nitinol spring/E chain for retraction, while the study group employed IZC screw-assisted en masse distalization. Regular reviews and adjustments occurred, with radiographs and study models assessed after six months for cephalometric parameters and arch length.

Results

A significant difference was found in U1-SN (degree), L1-Apog (in mm), L1-NB (degree), and L1-NB (in mm) of pretreatment records, whereas all other measurements showed statistically similar values between conventional and IZC groups. Improvement was higher with the conventional group when compared with IZC groups in these measurements due to the extraction of the first premolars rather than third molar extraction and distalization. However, the IZC group also showed statistically significant improvement in cephalometric parameters such as U1-SN (degree), L1-Apog (in mm), L1-NB (degree), and L1-NB (in mm).

Conclusion

The statistical analysis of radiographic and cast measurements in both the maxilla and mandible demonstrated a significant efficiency of IZC screws in teeth retraction while preserving arch length compared to conventional methods. Nevertheless, to strengthen the findings of our study, additional clinical investigations on IZC screws are warranted.

## Introduction

Precise control of three-dimensional tooth movement is essential in orthodontic treatment to avoid adverse effects resulting from applied forces. In instances of class 2 malocclusion or protrusion of teeth within the jawbone, the conventional approach involves extracting both the upper first premolars [1]. Subsequently, the plan is to retract the front teeth while ensuring maximum anchorage. Historically, extraction therapy or methods such as extra-oral and intra-oral maxillary molar distalization were employed in these cases. However, these methods often led to unintended reactionary forces, causing splaying and forward displacement of the front teeth [2-4].

En masse retraction, as conventionally practiced, may result in the extrusion of the upper front teeth, making it less suitable for individuals with vertical growth, deep overbite, or a prominent display of gums, potentially leading to unfavorable outcomes [5]. To address these issues, contemporary techniques like maxillary arch distalization using mini-implants have demonstrated greater effectiveness, yielding satisfactory results [6]. The incorporation of temporary anchorage devices (TADS), including mini-implants, micro-implants, and mini-screws (MS), has become integral to modern orthodontic approaches [7]. Mini-implants offer advantages such as multiple sites for the implant, smaller size, easy placement without extensive flap retraction, better patient compliance, immediate loading without the need for laboratory work, simplified posttreatment removal, and reduced overall expense [8].

The infrazygomatic crest (IZC) in the maxilla is an optimal site for implant placement due to its dense cortical plate, safer buccal location away from the apex of the roots, and flexibility enabling elevated positioning in the maxillary vestibule and facilitating unobstructed tooth movement during retraction in a single phase [9]. This contributes to an impressive 93.7% success rate for IZC implants [10]. Precision in anterior teeth movement is vital, and the use of power arms empowers orthodontists to achieve exact positioning by applying controlled force from the MS, enabling displacement and rotation in the sagittal and vertical planes during retraction [11].

Consequently, evaluating the effectiveness of IZC implants in the retraction of maxillary and mandibular teeth has become a topic of interest among clinicians and researchers. Hence, this study was aimed at comparing the cephalometric parameters of both pre and post treatment using IZC/buccal shelf (BS) screws and the conventional method of retraction and at assessing the efficiency of IZC screws in the retraction of teeth while maintaining the arch length.

## Materials and methods

A split-mouth study was undertaken with 40 patients at the Department of Orthodontics of Yenepoya Dental College in Mangalore, India. All subjects gave their informed consent for inclusion before they participated in the study. The study was conducted following the Declaration of Helsinki, and the protocol was approved by the Ethical Committee of Yenepoya Dental College (approval number: YEC-1/2019/210).

The following materials were used for the study: (1) orthodontic brackets, (2) orthodontic wires (0.016 NiTi, 16x22 NiTi, 19x25 NiTi, 17x25 SS, 19x25 SS), (3) crimpable hooks, (4) IZC/BS screws, (5) nitinol springs, (6) routine radiographic imaging, and (7) study models.

Case selection was based on class 2 malocclusion. Patients with class 2 malocclusion, who were willing to go for first premolar extraction, were taken in the control group. The ones willing for third molar extraction were taken in the study group. The participants were categorized into two groups based on their retraction method. The control group underwent a treatment plan comprising premolar extraction followed by anterior teeth retraction, while the study group's plan involved the removal of the third molars and the distalization of the entire arch using IZC screws.

The inclusion criteria include (1) patients within the age group of 18-35 years, (2) patients with class 1 malocclusion with bidental proclination, (3) patients with class 2 malocclusion requiring upper first premolar extraction for the correction of malocclusion, and (4) patients willing to give consent. In contrast, patients with poor oral hygiene, who were periodontally compromised, and with smoking habits were excluded from the study.

Participants were described about the study, and consent was also taken from them. Pretreatment records such as case history, extra-oral photographs, intra-oral photographs, impressions, and lateral cephalograms were recorded. The treatment plan decided the allotment of participants into control and study groups. Treatment for the control group commenced with the extraction of the premolar teeth and bonding of the upper and lower teeth with orthodontic brackets. After initial aligning and leveling of the teeth, retraction of the anterior teeth was carried out using the conventional technique, i.e., using a nitinol spring/E chain. Treatment for the study group included initial leveling and aligning, extraction of the third molars (when present), placement of IZC screws, and retraction using closed coil springs/E chain.

Just before the retraction phase of treatment, lateral cephalograms were taken for the participants followed by impressions of the upper and lower arch to analyze study models. All 20 participants were reviewed approximately every four weeks by the principal investigator. Both groups were bonded with a standard 0.022-inch McLaughlin, Bennett, and Trevisi (MBT) bracket system. The wires were incrementally proceeded, i.e., 0.014, 0.016, 0.018, 16x22 inch, 17x25 inch, and 19x25 inch, all made of NiTi, and 19x25 SS. On reaching 19x25 SS wire retraction phase was initiated. In the control group, the spring/E chains were attached from the first molar teeth to the crimpable hooks placed between the lateral incisor and canine on both sides of the 19x25 SS archwire.

In the study group, en masse distalization was carried out using IZC screws. Topical anesthesia (2% lignocaine) was sprayed above the IZC screw placement site, and then after a few minutes, IZC screws of dimension 2x12 mm were placed in the infrazygomatic (IZ) region in the maxilla on both sides. IZC screws were inserted in the IZ region at a height of 14-16 mm above the maxillary occlusal plane at the maxillary first molar region. The initial entry was at an angle of 90°; after a couple of turns, angulation was changed from 55° to 70° to the maxillary occlusal plane so that the roots of the molars do not suffer from damage. Optimal orthodontic forces were given from the IZC screws to the crimpable hooks placed in the region between lateral incisors and canines through closed coil springs/E chains.

**Figure 1:**
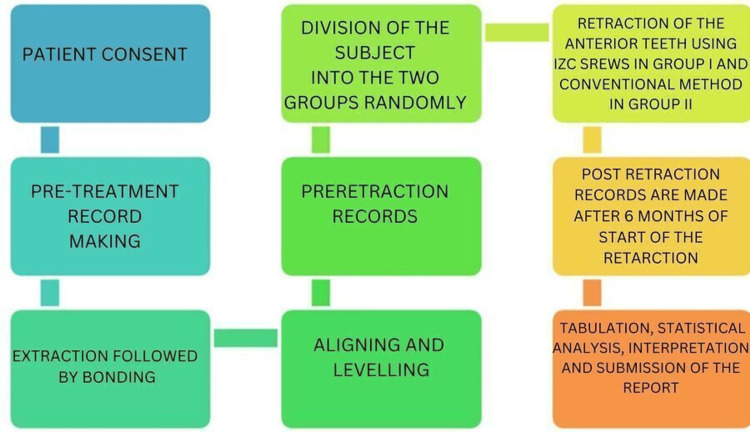
Clinical study design and plan

Six months after the start of the retraction, radiographs of the subjects were taken, and study models were made. On the lateral cephalogram, the following parameters were used for comparison: U1-SN, U1-NA (angular), U1-NA (linear), L1-Apog, L1-NB (angular), L1-NB (linear), U6 to Ptm, and upper lip to E-line and lip strain. Figure 1 explains the clinical study design and plan that we followed.

The study models were assessed for arch length as follows: The arch length was measured from a perpendicular line drawn from the center of the two central incisors to a horizontal line interconnecting the mesial fossa of the first molars in the same arch.

## Results

The cephalometric measurements and the cast measurements of both pre and post treatment of IZC and conventional groups were tabulated. The mean difference between the conventional and IZC groups was compared using an independent t-test.

Table 1 shows the arch length measurement on the maxillary cast before and after treatment with IZC which showed no significant difference between pre- and post-arch length measurement of the maxilla when retracted with IZC. 

**Table 1:** The arch length measurement (in mm) on the maxillary cast before and after treatment with IZC implants IZC: infrazygomatic crest

Arch length maxilla (in mm)		Mean	Standard deviation	P-value	95% confidence interval	
					Lower	Upper
	Pre	29.555	1.943	0.195	-0.210	0.876
	Post	29.222	1.641			

Table 2 shows the arch length measurement on the mandibular cast before and after treatment with IZC which showed no significant difference between pre- and post-arch length measurement of the mandible when retracted with IZC. 

**Table 2:** The arch length measurement on the mandibular cast before and after treatment with IZC implants IZC: infrazygomatic crest

Arch length mandible (in mm)		Mean	Standard deviation	P-value	95% confidence interval	
					Lower	Upper
	Pre	25.777	1.787	0.084	-0.189	2.411
	Post	24.666	3.201			

**Figure 2:**
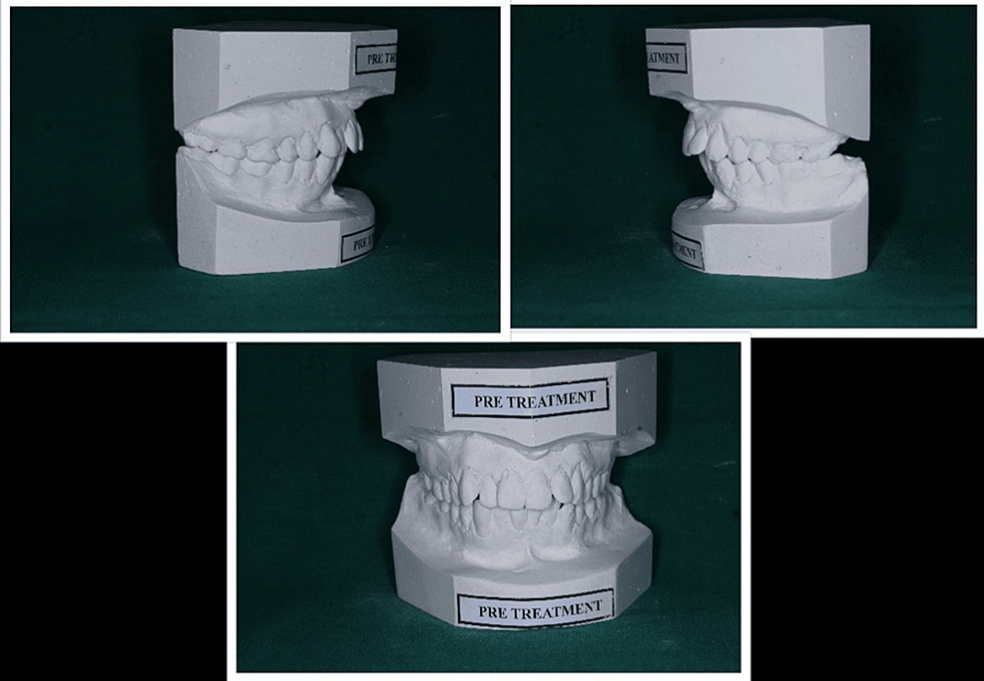
Pretreatment models showing lateral and anterior view in occlusion

Figure 2 shows the pretreatment models with lateral and anterior views in occlusion.

**Figure 3:**
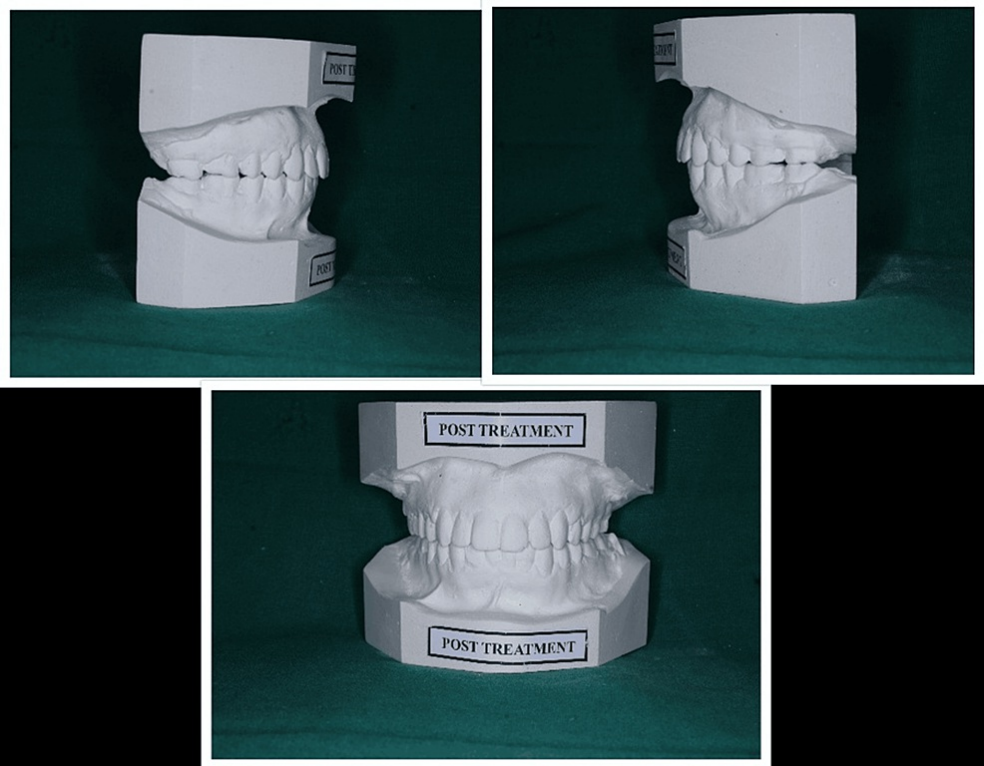
Posttreatment models showing lateral ad anterior views in occlusion

Figure 3 shows the posttreatment models with lateral and anterior views in occlusion.

Table 3 shows the radiographic measurements before treatment between the conventional and IZC groups which showed a significant difference in U1-SN (degree) of pretreatment records, whereas all other measurements showed statistically similar values between the conventional and IZC groups.

**Table 3:** The radiographic measurements before treatment between the conventional and IZC groups IZC: infrazygomatic crest

			Mean	Standard deviation	Test statistic	Mean difference	P-value	95% confidence interval	
								Lower	Upper
Pre-radiographic measurement	U1-SN (degree)	Case	114.666	3.122	-2.937	-5.666	0.010	-9.756	-1.576
		Control	120.333	4.873					
	U1-NA (degree)	Case	32.222	6.476	-1.364	-4.000	0.191	-10.216	2.216
		Control	36.222	5.953					
	U1-NA (in mm)	Case	10.555	4.216	0.519	0.777	0.611	-2.399	3.955
		Control	9.777	1.563					
	L1-Apog (in mm)	Case	8.444	3.876	0.737	1.000	0.472	-1.875	3.875
		Control	7.444	1.236					
	L1-NB (degree)	Case	35.666	5.024	-1.245	-2.777	0.231	-7.506	1.950
		Control	38.444	4.419					
	L1-NB (in mm)	Case	11.777	2.438	1.202	1.111	0.247	-0.849	3.071
		Control	10.666	1.322					

**Figure 4:**
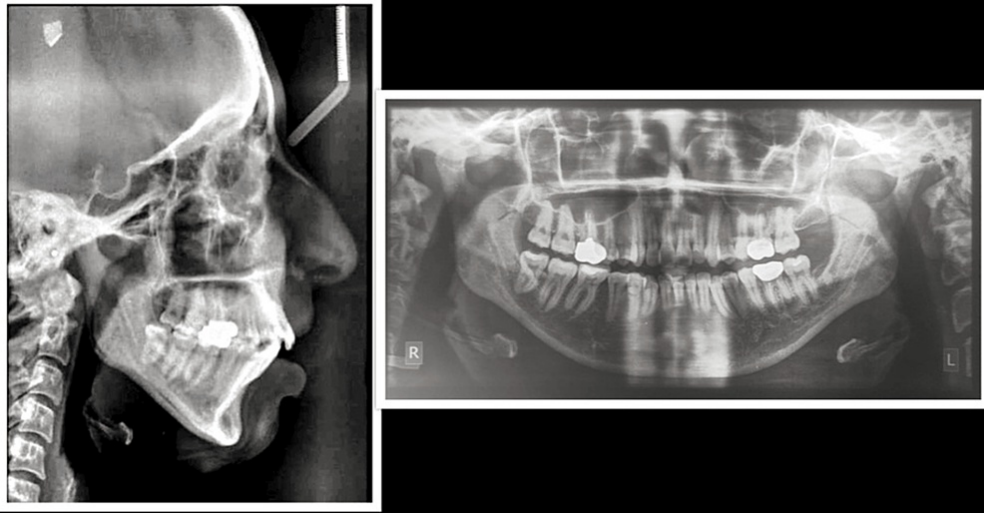
Pretreatment radiographs U1-SN: 114.666 degree; L1-Apog: 8.444 mm; L1-NB: 35.666 degree; L1-NB: 11.777 mm

Figure 4 shows the pretreatment radiographs of the patient.

Table 4 showed the radiographic measurements after treatment between the conventional and IZC groups which showed a significant difference in U1-SN (degree), L1-Apog (in mm), L1-NB (degree), and L1-NB (in mm) of pretreatment records, whereas all other measurements showed statistically similar values between the conventional and IZC groups.

**Table 4:** The radiographic measurements after treatment between the conventional and IZC groups IZC: infrazygomatic crest

			Mean	Standard deviation	Test statistics	Mean difference	P value	95% confidence interval	
								Lower	Upper
Post-radiographic measurements	U1-SN (degree)	Case	112.777	4.146	2.268	5.222	0.038	0.340	10.103
		Control	107.555	5.525					
	U1-NA (degree)	Case	27.333	6.224	0.357	1.000	0.726	-4.933	6.933
		Control	26.333	5.563					
	U1-NA (in mm)	Case	6.777	3.527	1.441	1.777	0.169	-0.837	4.392
		Control	5.000	1.118					
	L1-Apog (in mm)	Case	6.000	2.738	2.665	2.555	0.017	0.522	4.588
		Control	3.444	0.881					
	L1-NB (degree)	Case	30.777	4.841	2.736	4.777	0.015	1.075	8.479
		Control	26.000	2.000					
	L1-NB (in mm)	Case	9.000	1.500	5.149	3.444	0.001	2.026	4.862
		Control	5.555	1.333					

**Figure 5:**
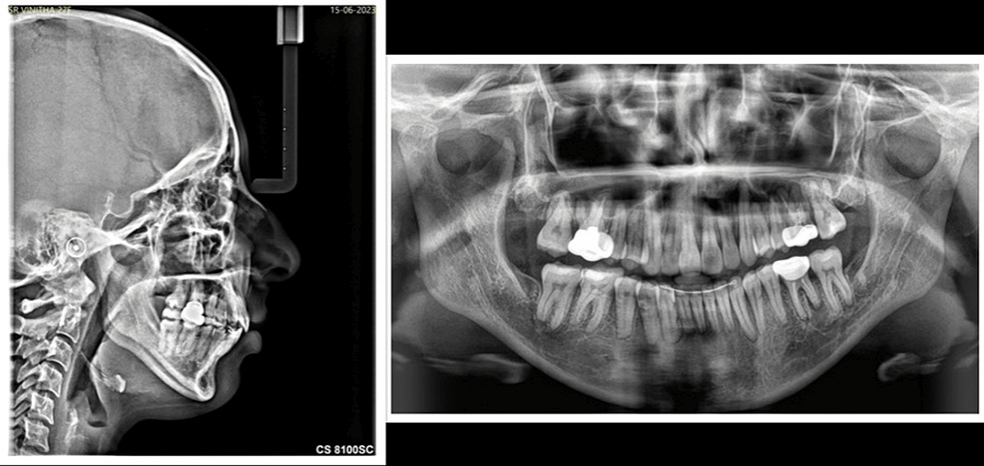
Posttreatment radiographs U1-SN: 112.777 degree; L1-Apog: 6 mm; L1-NB: 30.777 degree; L1-NB: 9 mm

Figure 5 shows the posttreatment radiographs of the patient.

Table 5 shows the comparison of radiographic measurements before treatment and after treatment with the IZC group. This showed a statistically significant difference between pre and post treatment for U1-SN (degree), L1-Apog (in mm), L1-NB (degree), and L1-NB (in mm) in the IZC group.

**Table 5:** Comparison of the radiographic measurements before and after treatment with the IZC group IZC: infrazygomatic crest

Radiographic measurement			Mean	Standard deviation	P-value	95% confidence interval	
						Lower	Upper
	U1-SN (degree)	Pre	114.666	3.122	0.113	-0.555	4.333
		Post	112.777	4.146			
	U1-NA (degree)	Pre	32.222	6.476	0.001	3.238	6.539
		Post	27.333	6.224			
	U1-NA (in mm)	Pre	10.555	4.216	0.006	1.447	6.108
		Post	6.777	3.527			
	L1-Apog (in mm)	Pre	8.444	3.876	0.002	1.222	3.666
		Post	6.000	2.738			
	L1-NB (degree)	Pre	35.666	5.024	0.093	-1.020	10.798
		Post	30.777	4.841			
	L1-NB (in mm)	Pre	11.777	2.438	0.001	1.458	4.096
		Post	9.000	1.500			

Table 6 shows the comparison of radiographic measurements before treatment and after treatment with the conventional group. This showed a statistically significant difference between pre and post treatment for U1-SN (degree), U1-NA (degree, mm), L1-Apog (in mm), L1-NB (degree), and L1-NB (in mm) in the IZC group. Improvement was higher with the conventional group when compared with the IZC group in these measurements.

**Table 6:** Comparison of the radiographic measurements before and after treatment with the conventional group

Radiographic measurement			Mean	Standard deviation	P-value	95% confidence interval	
						Lower	Upper
	U1-SN (degree)	Pre	120.333	4.873	0.001	9.806	15.749
		Post	107.555	5.525			
	U1-NA (degree)	Pre	36.222	5.953	0.001	7.600	12.177
		Post	26.333	5.634			
	U1-NA (in mm)	Pre	9.777	1.563	0.001	3.937	5.617
		Post	5.000	1.118			
	L1-Apog (in mm)	Pre	7.444	1.236	0.001	2.912	5.087
		Post	3.444	0.881			
	L1-NB (degree)	Pre	38.444	4.419	0.001	9.591	15.297
		Post	26.000	2.000			
	L1-NB(in mm)	Pre	10.666	1.322	0.001	4.062	6.159
		Post	5.555	1.333			

## Discussion

Orthodontic treatment faces a persistent challenge in achieving effective anchorage and influencing treatment plans and results. Different anchorage methods, such as extra-oral anchorage, opposing anchors, and increasing the number of teeth in anchor units, have been suggested. However, these approaches frequently carry undesired side effects and are dependent on patient compliance. Extra-oral anchorage, in particular, is challenging to use and may result in injuries that affect patient adherence [9].

Kanomi introduced MS as TADs to address patient compliance challenges, particularly for retracting anterior teeth [12]. However, interradicular (IR) MS, placed between roots, poses issues such as high failure rates, interference with tooth movement, and impingement on adjacent roots [13-15].

Ample extra-alveolar sites offer TAD placement such as the premaxillary and mid-palatal region, canine and incisive fossa, anterior external oblique ridge (AEOR), retromolar area, and sublingual fossa. This study focuses specifically on the IZ region as an extra-alveolar site.

The IZC in the maxilla has shown success as an extra-alveolar site for skeletal anchorage in orthodontic procedures, such as maxillary canine retraction and anterior retraction. This study aims to assess the effectiveness of IZC screws in tooth retraction while preserving arch length, comparing it with conventional retraction methods.

Research by Sanap et al. [16] and Paul et al. [17,18] highlighted the efficacy of IZC screws in maxillary arch distalization and stress distribution at different angles and forces. Bechtold et al. reported on the intrusion of the entire dental arch using dual IR MS in the maxilla, impacting the arch's relationship with the line of force [19]. Shaik et al. examined the effectiveness of IZ implants for full-arch distalization and reducing gummy smiles in class 2 division 1 malocclusion patients, revealing significant clinical and statistical outcomes [9].

In our study, a significant difference was found in U1-SN (degree), L1-Apog (in mm), L1-NB (degree), and L1-NB (in mm) of pretreatment records, whereas all other measurements showed statistically similar values between the conventional and IZC groups. Improvement was higher with the conventional group when compared with the IZC group in these measurements due to the extraction of the first premolars rather than third molar extraction and distalization. However, the IZC group also showed statistically significant improvement in cephalometric parameters such as U1-SN (degree), L1-Apog (in mm), L1-NB (degree), and L1-NB (in mm).

Complications associated with IZC implants are infrequent. One must consider removing fully erupted third molars in order to create space aiding the distalization process. Extraction is not needed for distalization when unerupted third molars are situated below the cementoenamel junction of the second molars in younger individuals. The primary concerns related to bone screws include gingival overgrowth and early loosening, with larger-headed screws showing reduced overgrowth. In the event of loosening, implants can be replaced at different sites.

Limitations of the study

It is important to note that the study has limitations, including a short-term evaluation, a small study population, and the potential influence of growth during treatment.

## Conclusions

The efficiency of IZC screws in the retraction of teeth while maintaining the arch length and their effectiveness in en masse distalization were statistically significant with the conventional methods as revealed by radiographic measurements and cast measurements in both the maxilla and mandible. However, further clinical studies have to be done on IZC screws to substantiate the results of our study.
